# Emerging markers of cachexia predict survival in cancer patients

**DOI:** 10.1186/1471-2407-14-828

**Published:** 2014-11-16

**Authors:** Patrizia Mondello, Antonio Lacquaniti, Stefania Mondello, Davide Bolignano, Vincenzo Pitini, Carmela Aloisi, Michele Buemi

**Affiliations:** Department of Human Pathology, University of Messina, Via Consolare Valeria, 98125 Messina, Italy; Department of Internal Medicine, University of Messina, Messina, Italy; Department of Neurosciences, University of Messina, Messina, Italy; CNR-Institute of Clinical Physiology, Reggio Calabria, Italy

**Keywords:** Ghrelin, Leptin, Obestatin, Cancer cachexia, Survival, Biomarkers

## Abstract

**Background:**

Cachexia may occur in 40% of cancer patients, representing the major cause of death in more than 20% of them. The aim of this study was to investigate the role of leptin, ghrelin and obestatin as diagnostic and predictive markers of cachexia in oncologic patients. Their impact on patient survival was also evaluated.

**Methods:**

140 adults with different cancer diagnoses were recruited. Thirty healthy volunteers served as control. Serum ghrelin, obestatin and leptin were tested at baseline and after a follow-up period of 18 months.

**Results:**

Ghrelin levels were significantly higher in cancer patients than in healthy subjects (573.31 ± 130 vs 320.20 ± 66.48 ng/ml, p < 0.0001), while obestatin (17.42 ± 7.12 vs 24.89 ± 5.54 ng/ml, p < 0.0001) and leptin (38.4 ± 21.2 vs 76.28 ± 17.48 ng/ml, p < 0.0001) values were lower. At ROC analyses the diagnostic profile of ghrelin (AUC 0.962; sensitivity 83%; specificity 98%), obestatin (AUC 0.798; sensitivity 74.5%; specificity 81.5%) and leptin (AUC 0.828; sensitivity 79%; specificity 73%) was superior to that of albumin (AUC 0.547; sensitivity 63%, specificity 69.4%) for detecting cachexia among cancer patients. On Cox multivariate analyses ghrelin (HR 1.02; 95% CI 1.01 – 1.03; p < 0.0001) and leptin (HR 0.94; 95% CI 0.92 – 0.96; p < 0.0001) were significant predictors of death even after correction for other known risk factors such as presence of metastasis and chronic kidney disease.

**Conclusion:**

Ghrelin and leptin are promising biomarkers to diagnose cachexia and to predict survival in cancer patients.

## Background

Weight loss is commonly observed in several types of cancer, and poor nutritional status has been associated with decreased survival and poorer quality of life in the cancer population [1–3].

Cachexia may occur in 15% to 40% of patients with cancer and in about 80% of patients with advanced illness, representing the major cause of death in more than 20% of patients [4, 5].

According to a consensus panel, cachexia should be diagnosed when, in the presence of an underlying disease, there is a weight loss of at least 5% in 12 months or less associated with any 3 of the following criteria: decreased muscle strength, fatigue, anorexia, low fat-free mass index, increased inflammatory markers such as C Reactive Protein (CRP >5.0 mg/L) or interleukin (IL)-6 (>4.0 pg/ml), anemia (Hb <12 g/dl) or low serum albumin (<3.2 g/dl) [6].

To date, even though clinical practice guidelines are available [7–9] and provide recommendations for prevention and treatment of cancer-related anorexia cachexia syndrome (CACS), the management of CACS is still a complex challenge. Biomarkers that can accurately diagnose CACS in the early stage and predict progression and outcome would have important implications for developing the most effective interventions.

Food intake and energy homeostasis are regulated by a complex network of peripheral mediators, such as hormones, neuropeptides, and cytokines. In particular, inflammatory cytokines and other hormonal factors have been postulated to play a role in the development of cachexia [10, 11]. Therefore previous studies have evaluated the utility of albumin and prealbumin as markers of malnutrition and shorter survival, even if they have shown low sensitivity and specificity [12–15].

Similarly, high levels of inflammatory markers and low hemoglobin value, which are usually associated with increased mortality, do not significantly improve the ability to predict survival better than cancer stage, albumin, and weight loss [12, 16].

Leptin, primarily produced by adipocytes in proportion to body fat, has been postulated to play a major role in the pathophysiology of cancer cachexia [17]. Decreased levels of leptin have been demonstrated in cancer patients with cachexia compared to those without cachexia and healthy controls [18].

Ghrelin and obestatin, two gastrointestinal peptides obtained by post-translational processing of preproghrelin, are part of a complex gut-brain network informing the brain about satiety or hunger [19]. Plasma ghrelin levels are generally inversely related to body mass index (BMI). Neary et al. demonstrated that the administration of ghrelin in cancer patients with severe anorexia resulted in a marked increase in both food consumption and energy intake compared to controls [20]. Conversely, obestatin appears to act as an anorectic hormone, decreasing food intake and reducing body weight gain [21, 22]. Obestatin showed to be correlated positively with ghrelin and negatively with BMI [23], insulin [24] and serum level of vasopressin [25]. This suggests that basal secretions of obestatin and ghrelin may be regulated in a similar manner, since they are both influenced by adiposity and insulin resistance [26].

Based on previous research and because of the evidence of the important role played in the homeostasis of body weight regulation, we hypothesized that leptin, ghrelin and obestatin could be useful as diagnostic markers of cachexia in oncologic patients. We also evaluated their relation to patient survival.

## Methods

### Patients and controls

Between February 2010 and January 2011, we prospectively enrolled 140 adult oncologic patients (74 men: mean age 57.7 ± 11.3 years; 66 women, mean age: 60.6 ± 12 years) admitted to the oncology unit of the University Hospital “G. Martino”, Messina, Italy.

Exclusion criteria were: physician-recorded diagnosis of formally evaluated dysphagia (by speech pathologist otolaryngologists), illicit drug or alcohol abuse, severe congestive heart failure (defined as New York Heart Association (NYHA) class III or IV), abnormal liver function, severe chronic obstructive pulmonary disease, uncontrolled diabetes (fasting glucose levels of >140 mg/dL or random glucose levels of >200 mg/dL), thyroid disease (defined as abnormal levels of thyroid hormone levels causing hypo- or hyperthyroidism condition), severe kidney disease (defined as glomerular filtration rate <15 ml/min), active infection, history of neuroendocrine tumor, diagnosed eating disorders or use of orexigenic agents (e.g., chronic use of glucocorticoids, progesterone, testosterone, and antiandrogens).

Diagnosis of cachexia follows the definition reported by Evans [6]. In particular we considered as cachexia the presence of a weight loss of at least 5% or more in 12 months or less, plus BMI <20, fatigue, abnormal biochemistry (increased inflammatory markers: CRP >5.0 mg/L), anemia (<12 g/dL) condition and low serum albumin (<3.2 g/dL).

Demographic and other data regarding the type of cancer and cachexia diagnosis, cancer stage, treatment, comorbidity, were also recorded. To assess percentage of weight change, patients’ body weight was evaluated at enrollment (baseline) and at 18 months. If the subject died before the end of follow up, the last weight recorded was used to calculate the percent of weight change. Common biochemical parameters including urea, creatinine, uric acid, serum lipids, total serum calcium, phosphorus, calcium-phosphate product, serum iron, electrolytes, albumin, hemoglobin, total alkaline phosphatases and fibrinogen were measured at baseline in all patients and controls. Survival was determined at 18 months.

As a control group, 30 healthy volunteers without clinical history of cancer and cardiovascular or metabolic diseases were recruited.

The study was approved by the Province of Messina Ethics Committee and fully informed consent was obtained in writing from all participants.

### Collection of blood and Ghrelin, obestatin and leptin dosage

In the patient group and in the healthy control group blood samples were collected at 08:00 h after an overnight fast.

Blood samples were collected into chilled vacutainer tubes containing potassium ethylenediamine tetracetate. Tubes were instantly cooled on ice and centrifuged at 3000 rpm for 10 min at 4°C within 30 min, and aliquots were immediately stored at −80°C until analyzed.

Serum ghrelin, obestatin and leptin were tested using an enzyme-linked immunosorbent assay (ELISA) commercial available kit (Obestatin: Bachem Distribution Services GmbH, Weil am Rhein, Germany; Leptin: R&D Systems Space Import-Export srl Milan, Italy; Ghrelin: Phoenix Europe GmbH, Germany) according to the manufacturer’s instructions. The enzymatic reactions were quantified in an automatic microplate photometer.

### Statistical analysis

Statistical analyses were performed with NCSS for Windows (version 4.0), the MedCalc (version 11.0; MedCalc Software Acacialaan, Ostend, Belgium) software and the GraphPad Prism (version 5.0; GraphPad Software, Inc., San Diego, CA, USA) package. Data were presented as mean ± SD for normally distributed values (at Kolmogorov-Smirnov test) and median [IQ range] for non-normally distributed values. Differences between groups were established by unpaired t test for normally distributed values and by Kruskal-Wallis analysis followed by Dunn’s test for nonparametric values. Dichotomized values were compared using the *x*^2^ test. Receiver operating characteristics (ROC) analysis was employed to calculate the area under the curve (AUC) for ghrelin, obestatin, leptin and albumin and to find the best cut-off values able to identify the presence of cachexia.

Survival analyses used the Kaplan–Meier survival curve and the Cox proportional hazards model. Adjusted risk estimates for survival were calculated using univariate followed by multivariate Cox proportional hazard regression analysis. Exploratory graphical analysis and test of specific violations indicated no departure from the assumption of proportional hazards. All results were considered significant if p was <0.05.

## Results

### Study population

Demographic, clinical and laboratory data for controls and cancer patients are shown in Table 1. Patients and controls were well matched with regard to demographic characteristics. 28 patients were diabetic (20%) with good glycemic control (HbA1c 5.3 ± 1.6%). 37% of all patients were hypertensive, 16% had mild congestive heart failure (NYHA class I and II) and 56% had a chronic kidney disease. 105 patients (75%) were in the most advanced stage of cancer disease (namely stage IV according to the TNM and Ann Arbor staging systems for solid tumors and lymphomas respectively and stage III according to the International Staging System for multiple myeloma), whereas 35 (25%) belonged to stage III.

**Table 1:** **Baseline demographic, clinical and laboratory data of the study population**

Parameters	Cancer group	HS group	Follow up (18 months)		
			Death	Survivors	***P***
	n:140	n: 30	n:94 (67%)	n:46 (33%)	
Age, y	61.8 ± 14.3	59.6 ± 12.2	67.4 ± 6.3	60.7 ± 8.9	0.01
Weight change, %	12.1 ± 8.9	-	18.6 ± 1.8	7.6 ± 2.3	< 0.0001
Tot cholesterol (mg/dL)	142.9 ± 27.2	175.6 ± 12.6	139.6 ± 9.6	141.6 ± 10.6	0.23
Diabetics, *n*	28 (20%)	-	18	10	0.10^*^
Hypertension, *n*	52 (37%)	-	30	22	0.19^*^
Heart Failure, *n*	16 (11%)	-	10	6	0.29^*^
CKD, *n*	56 (40%)	-	37	19	0.003
Hemoglobin, g/dl	10.1 ± 2.5	13.6 ± 1.7	9.3 ± 1.6	10.8 ± 1.8	< 0.0001
Metastatic disease, *n*	105 (75%)	-	90	15	<0.0001^*^
Albumin, g/L	2.7 ± 0.6	4.02 ± 0.8	2.3 ± 0.4	2.5 ± 0.5	0.004
Obestatin, ng/ml	17.4 ± 7.1	24.8 ± 5.5	12.3 ± 2	15.1 ± 4.8	0.005
Leptin, ng/ml	38.4 ± 21.2	76.2 ± 17.4	21.8 ± 10	33.8 ± 9.1	< 0.0001
Ghrelin ng/ml	573.3 ± 130	320.2 ± 66.8	657.6 ± 88	481.4 ± 43.6	< 0.0001

*P* values calculated by using the *t* test, except where indicated.

^*^Calculated by using *x* ^2^ test.

*Abbreviations*: *HS* healthy subjects, *CKD* chronic kidney disease, defined as glomerular filtration rate <60 ml/min.

Enrolled patients were treated with one (28%) or more (61%) chemotherapy lines and/or surgery treatment (21%) and/or radiotherapy irradiations (17%). Oncologic and treatment characteristics of the 140 patients included in this study are summarized in Table 2.

**Table 2:** **Oncologic characteristics and chemotherapy drugs used**

Cancer type, n (%)	Surgery, n 30 (21)	Radiotherapy, n 25 (17)	Chemotherapy, n 129 (92)
Gastrointestinal cancer 27 (20%)	11 (37)	4 (16)	26 (21)
Lung and pleura cancer 25 (18%)	3 (10)	1 (4)	25 (19)
Pancreas cancer 3 (2%)	1 (3)	-	2 (1)
Breast cancer 20 (14%)	9 (30)	9 (36)	18 (14)
Multiple Myeloma 13 (9%)	-	5 (20)	12 (9)
Lymphomas 28 (20%)	-	4 (16)	25 (19)
Head and Neck cancer 16 (11%)	1 (3)	2 (8)	15 (12)
Gynecologic cancer 8 (6%)	5 (17)	-	7 (5)
Cancer Stage	Patients, n (%)	Death, n (%)	Survival, n (%)
III*	35 (25)	4 (5	31 (68%)
III**- IV*	105 (75)	90 (95%)	15 (32%)
Chemotherapy drugs			
Platinum	59 (42)	48 (81%)	11 (9%)
Anthracyclines	55 (39)	33 (60%)	22 (40%)
Taxanes	16 (12)	6 (37%)	10 (63%)
Anti-metabolites	75 (53)	57 (76%)	18 (24%)
Alkylating Agents	18 (13)	9 (50%)	9 (50%)
Vinca Alkaloids	16 (11)	7 (43%)	9 (57%)
Monoclonal Antibodies	24 (17)	13 (54%)	11 (46%)
Hormone therapy	12 (9)	6 (50%)	6 (50%)

*This stage refers to patients with solid tumors and lymphomas according to the TNM and Ann Arbor staging systems respectively.

**This stage refers to patients with multiple myeloma according to the International Staging System (ISS).

### Serum concentration of Ghrelin, obestatin and leptin

Ghrelin levels were significantly higher in cancer patients than in healthy subjects (HS) (573.31 ± 130 versus 320.20 ± 66.48 ng/ml, p <0.0001), while levels of obestatin and leptin were significantly lower (obestatin, 17.42 ± 7.12 versus 24.89 ± 5.54 ng/ml, p <0.0001; leptin, 38.4 ± 21.2 versus 76.28 ± 17.48 ng/ml, p <0.0001).

Accordingly with cancer stages, patients in stage IV showed the highest ghrelin levels (683.5 ± 73.4 ng/ml), the lowest leptin (19.67 ± 11.5 ng/ml) and obestatin values (9.2 ± 4.1 ng/ml) with respect to stage III. However, no differences in these three hormone levels were found to be related to the tumor burden, defined as number of metastatic sites. In particular, ghrelin levels observed in patients with one metastatic lesion (568.3 ± 51.2 ng/ml) were not statistically different from values observed in patients affected by three or more metastatic sites (556 ± 108.47 ng/ml; p:0.82).

### Univariate correlations

**Figure 1:**
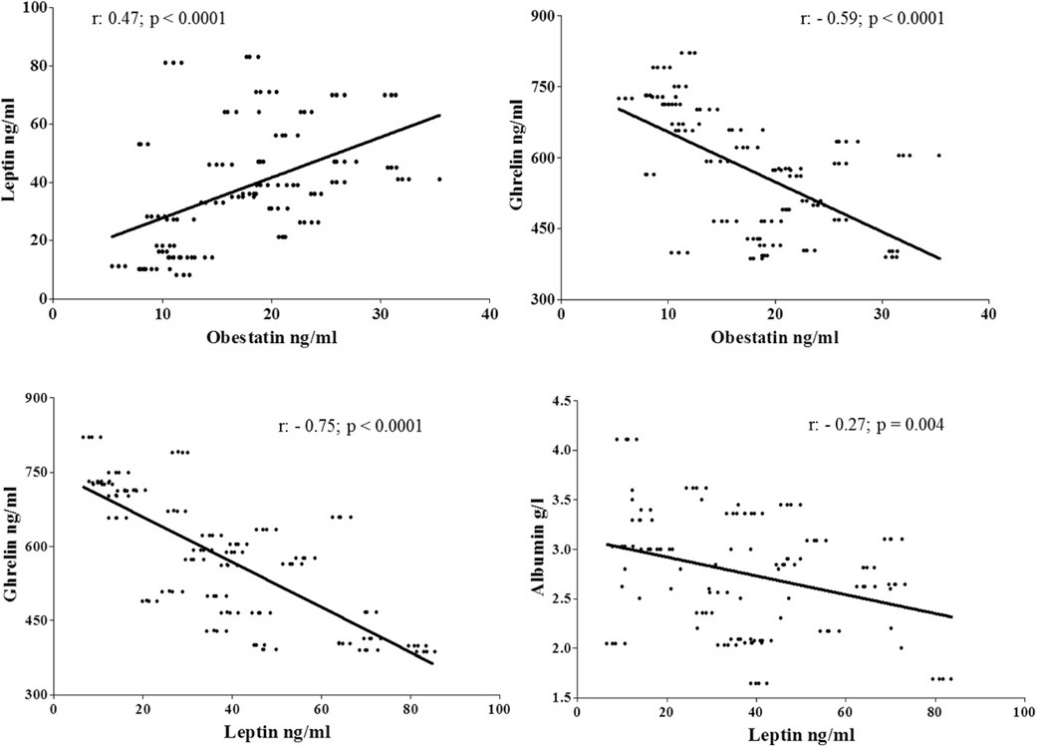
**Univariate relationships between ghrelin & obestatin and ghrelin & leptin.**

At univariate analysis, obestatin concentrations were positively correlated with leptin (r = 0.47; p <0.0001; Figure 1) age (r = 0.19 ; p = 0.01 ), heart failure (r =0.30; p = 0.003), and presence of metastasis (r = 0.39; p = 0.002), whereas a negative correlation with ghrelin (r = -0.59; p <0.0001; Figure 1), cholesterol (r = -0.45; p <0.0001), diabetes mellitus (r = -0.26; p = 0.002) and albumin (r = -0.21; p = 0.03) was found.

Ghrelin correlated positively with cholesterol (r 0.29; p = 0.004) and diabetes mellitus (r =0.17; p =0.03), and negatively with obestatin (r = -0.59; p <0.0001), leptin (r = -0.75; p <0.0001; Figure 1), heart failure (r = -0.25; p = 0.001), presence of metastasis (r = -0.35; p <0.0001) and serum protein levels (r = -0.33; p =0.0001).

Leptin concentrations were positively correlated with obestatin (r = 0.47; p <0.0001), presence of metastasis (r = 0.34; p <0.0001), and serum protein levels (r = 0.23; p = 0.01) whereas a significant inverse correlation was found with ghrelin (r = -0.75, p <0.0001), albumin (r = -0.27; p = 0.004; Figure 1) and cholesterol (r = -0.28; p = 0.006). Univariate relationships between leptin, obestatin, ghrelin and albumin are summarized in Figure 1.

### Multiple regression analysis

All variables found to be significantly correlated with obestatin at univariate analysis were introduced in a multivariate model using obestatin as the dependent variable. After adjustment for other factors, only the correlations between obestatin and albumin (β = -0.65; p = 0.0003), cholesterol (β = - 0.48; p = 0.004), age (β = 0.23; p = 0.0007) and metastasis (β = 0.47; p = 0.03) remained significant.

After adjustment for other factors, using ghrelin as dependent variable, only the correlation between ghrelin and cholesterol (β = 0.32; p = 0.02), and metastasis (β = - 0.43 ; p = <0.0001) attained significance.

Analyzing leptin as dependent variable in a multivariate model, significant correlation was maintained only between leptin and metastasis (β = 0.40; p <0.001).

### Diagnostic profile of obestatin, leptin and Ghrelin as biomarkers of cachexia

ROC analyses were performed in order to define the diagnostic profile of obestatin, leptin and ghrelin in identifying cachexia among cancer patients.

**Figure 2:**
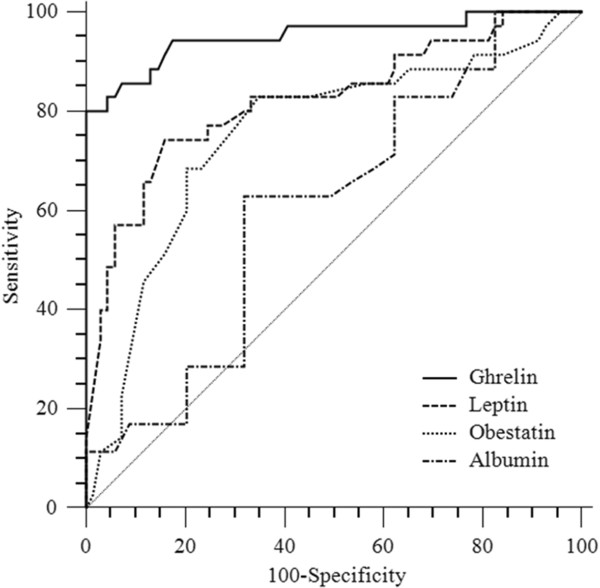
**Receiver operating characteristics (ROC) curves. ROC curves of ghrelin, leptin, obestatin and albumin in oncological subjects with cachexia.**

The area under the ROC curve for ghrelin, obestatin and leptin were 0.962, 0.798 and 0.828, respectively. Best cut-off values for ghrelin, obestatin and leptin were 663 (sensitivity 83%; specificity 98%), 13 (sensitivity 74.5%; specificity 81.5%) and 31 ng/ml (sensitivity 79%; specificity 73%), respectively. Conversely, the diagnostic profile of albumin was very poor (AUC 0.547; best cut-off value of 2.9 g/dl; sensitivity 63%, specificity 69.4%). AUC of ghrelin was statistically different compared with that of obestatin (p <0.001), leptin (p =0.003) and albumin (p <0.001). Obestatin and leptin areas were statistically different compared with that of albumin (p = 0.03; p = 0.003, respectively). On the contrary, there were no differences between obestatin and leptin areas (p >0.05) Figure 2.

### Ghrelin, leptin and obestatin: survival prediction

**Figure 3:**
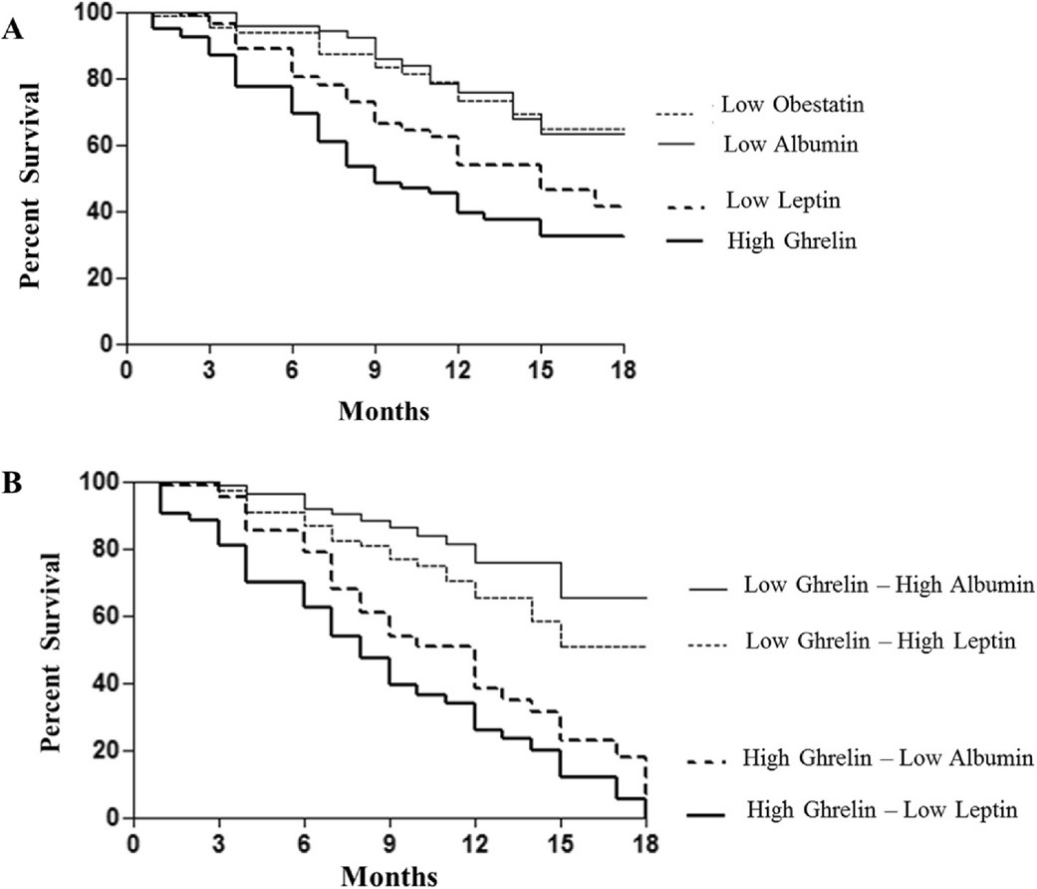
**Kaplan–Meier all-cause mortality curves. A** Kaplan-Meier survival curves of end-point (mortality during a median follow-up period of 18 months) in patients with ghrelin, leptin, albumin and obestatin levels above and below the optimal receiver operating characteristics cut-off level. Patients with ghrelin >663 ng/ml showed a significantly faster progression to endpoint (log-rank (χ^2^) 5.02; p = 0.02) **B** Association of ghrelin, leptin or albumin to provide the best predictive model of mortality. Patients with high levels of ghrelin and low leptin levels were characterized by the worst outcome (log-rank (χ^2^) 8.02; p = 0.004). Patients with low levels of ghrelin and high levels of albumin instead had the best profile, although there were no statistically significant differences if compared with patients with low levels of ghrelin and high levels.

Biomarkers of cachexia were tested by Kaplan Meier analysis, alone or in combination, with respect to all cause-mortality during a median follow-up period of 18 months. During this period, 94 (67%) deaths occurred. Kaplan–Meier analysis showed that patients with ghrelin levels above the optimal ROC-derived cut-off value had a poorer survival (log-rank (χ^2^): 5.02; p = 0.02), as well as those with leptin values below 31 ng/ml (log-rank (χ^2^): 3.24; p = 0.03) and obestatin values below 13 ng/ml (log-rank (χ^2^): 2.86; p = 0.03) with a mean follow-up time of 16 months and 17 months respectively (Figure 3A).

High levels of ghrelin and low leptin values were associated with minor survival probability with respect to those observed in patients characterized by high levels of ghrelin and low values of albumin. (log-rank (*x*^2^) 8.02; p = 0.004).

On the contrary, patients with the best profile were those with low levels of ghrelin associated with high levels either of albumin or leptin, without any statistical difference between these two combinations (log-rank (χ^2^): 2.60; p = 0.10) Figure 3B.

### Univariate/multiple Cox regression analysis

To identify putative risk factors associated with all-cause mortality we performed a Cox regression analysis by inserting in the model all variables that were different at baseline between survivors and non-survivors Table 1.

At univariate analysis, only ghrelin (HR 1.01; 95% CI 1.00 – 1.01; *x*^2^: 2.87; p 0.005), obestatin (HR 1.13; 95 CI 1.03 – 1.24; *x*^2^: 3.71; p 0.009), leptin (HR 0.92; 95% CI 0.88 – 0.95; *x*^2^: 5.36; p 0.0006), the presence of metastatic lesions (HR 1.15; 95% CI 1.25 – 8.02; *x*^2^: 2.12; p 0.01) and chronic kidney disease (HR 2.19; 95% CI 1.11 – 4.34; *x*^2^: 2.37; p 0.02) were significantly associated with the endpoint, whereas body weight decrease, hemoglobin, albumin and age failed to reach statistical significance.

**Figure 4:**
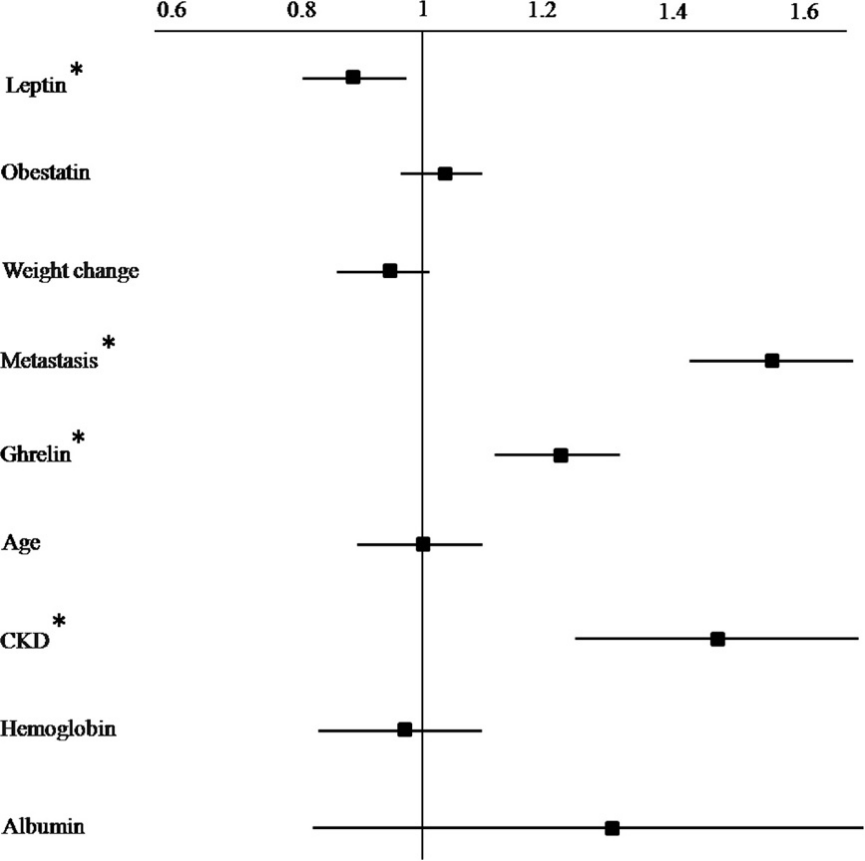
**Cox proportional hazard regression models including the effects of different variables on survival.** CKD: chronic kidney disease. Weight change was measured from baseline to 18 months follow-up.

In a multiple Cox regression model, all the variables found to be significantly associated with the endpoint at univariate analysis (ghrelin, obestatin, leptin, metastatic disease and chronic kidney disease) were tested to identify independent predictors (Figure 4). In addition, age was also inserted in this model as it commonly represents one of the most important risk factors for death.

Results from this analysis indicated that ghrelin (HR 1.02; 95% CI 1.01 – 1.03; *x*^2^: 5.81; p < 0.0001), leptin (HR 0.94; 95% CI 0.92 – 0.96; *x*^2^: 4.65; p 0.0001), the presence of metastatic disease (HR 2.67; 95% CI 1.45 – 4.91; *x*^2^: 3.86; p 0.001) and chronic kidney disease (HR 1.97; 95% CI 1.24 – 3.12; *x*^2^: 2.14; p 0.003), remained significantly associated with mortality.

Table 3 summarizes data from univariate and multivariate Cox regression analysis.

**Table 3:** **Univariate and multivariate Cox proportional hazards regression model for death**

	Univariate analysis				Multivariate analysis			p value
	HR	95% CI	***x*** ^2^	p value	HR	95% CI	***x*** ^2^	
Leptin	0.92	0.88 – 0.95	5.36	0.0006	0.94	0.92 – 0.96	4.65	0.0001
Ghrelin	1.01	1.00 – 1.01	2.87	0.005	1.02	1.01 – 1.03	5.81	< 0.0001
Obestatin	1.13	1.03 – 1.24	3.71	0.009	1.04	0.99 – 1.10	1.05	0.10
Albumin	1.66	0.81 – 3.40	1.23	0.16				
CKD	2.19	1.11 – 4.34	2.37	0.02	1.97	1.24 – 3.12	2.14	0.003
Metastasis	1.15	1.25 – 8.02	2.12	0.01	2.67	1.45 – 4.91	3.86	0.001
Age	1.00	0.95 – 1.04	0.16	0.95	1.00	0.95 – 1.01	0.15	0.93
Weight change	0.90	0.82 – 1.00	0.27	0.90				
Hemoglobin	0.99	0.86 – 1.15	0.12	0.97				

HR: hazard ratio; CI: confidence interval; CKD: chronic kidney disease.

Weight change was measured from baseline to 18 months follow-up.

## Discussion

We demonstrated that ghrelin and leptin represent promising biomarkers for the identification of cachexia associated with cancer.

In accordance with previous observations [27], we found that ghrelin levels were higher in the cachexia group compared to the control group. The increased concentration of ghrelin in oncologic patients may represent a compensatory mechanism for catabolic-anabolic imbalance, which is unable to reverse despite suppression of food intake and muscle wasting [28]. We also found a strict inverse correlation between ghrelin and heart disease, confirming the existing data on the cardio-protective role of this hormone [29]. In addition, ghrelin was correlated with the presence of diabetes mellitus. Previous studies showed that ghrelin exerts modulatory action on insulin secretion and glucose metabolism [30], suggesting a negative association between ghrelin and insulin [31, 32].

Our oncologic patients were also characterized by lower levels of leptin and obestatin. Mantovani et al have already showed that circulating leptin concentrations are lower in anorectic cancer patients than in healthy individuals [17].

Proinflammatory cytokines, such as TNF-α, IL-1, and IL-6, have been proposed to cause cachexia despite low circulating leptin, due to the increased expression of the hypothalamic leptin receptor [33]. This deregulation of the physiological feedback may explain why a decrease in leptin does not increase appetite or lower energy expenditure in patients with cancer cachexia. There are several *in vitro* data on obestatin involvement in the oncologic field [21]. Different studies have shown that obestatin has a role in regulating the cell cycle by exerting proliferative effects through the inhibition of cell proliferation markers or by mitogen-activated kinase/extracellular signal-regulated kinases1/2 (ERK1/2) phosphorylation. Moreover, anti-proliferative effects have been assigned to obestatin. However, this is the first *in vivo* study in which obestatin has been related to cancer disease in humans. Our data confirmed that this hormone plays an antithetic role with ghrelin in energy balance, as demonstrated by the low levels of obestatin opposed to the high levels of ghrelin. The relationship between obestatin, cardiac dysfunction and diabetes might confirm its protective role on the cardiovascular system and the negative influence on metabolic control [34, 35]. Moreover, we found an independent correlation between ghrelin, obestatin, leptin and severity of cancer disease in our patients.

We have also shown that these three hormones hold a high diagnostic sensitivity and specificity for detecting cachexia in cancer patients. The hypothesis that nutrition-related variables predict survival was supported by our results. In particular, patients with higher ghrelin levels had a worse all-cause mortality, but if we consider the associations of the three biomarkers, patients with higher levels of ghrelin and lower leptin values were characterized by the worst outcome. We analyzed the effect of all variables that were different at baseline on survival. Ghrelin and leptin remained significant predictors of death independently of other potential confounders, such as the presence of metastasis or chronic kidney disease.

Our results support the inclusion of ghrelin and leptin among those factors able to influence survival in cancer patients. Furthermore, this study included non-terminally ill patients; this indicates that assessing these variables might also be useful in the non-terminal cancer population.

This study has a major limitation represented by the high heterogeneity of neoplasias and the severity of disease in patients enrolled in the study. Although ghrelin and leptin were strongly associated with survival in the whole study cohort, this heterogeneity could potentially hamper the reliability of these results. Moreover, it is possible that hormone levels could be influenced by chemotherapy.

## Conclusion

Our data suggest that simple and inexpensive testing may be helpful to assess an oncology patient’s long-term risk of mortality. Calculations of body weight change and the evaluation of emerging biomarkers of nutritional status may give the clinician a more accurate picture of the patient’s prognosis. Further prospective studies are necessary to confirm the potential application of these hormones and to ascertain their relevance as a parameter for monitoring the development of CACS and the progression of cancer disease.

In conclusion, there is a need to understand and explore the role of various neuropeptides and cytokines in the pathophysiology of cancer-anorexia syndrome so that therapeutic measures, such as ghrelin administration, may be designed to improve the survival of oncologic patients.

## Authors’ information

**Patrizia Mondello, MD**, graduated with mark 110/110 cum laude in Medicine and Surgery at the University of Messina (Italy) in 2009 defending an experimental thesis entitled “Primary Lymphoma of the central nervous system” and is currently attending the fifth year of the residency in Oncology. She pursues different cancer specializations, focusing on the hematoncology field. She has received many awards and spent long periods in Germany and the USA, furthering her medical and language knowledge. She is currently spending her second year at the Memorial Sloan Kettering Cancer Center in New York working in Dr Anas Younes’s laboratory as research fellow and furthering her knowledge of translational research in the lymphomas field. She is author and co-author of scientific papers published in national and international peer-review journals. Her main fields of research focus on pathophysiology in onco- and hematology fields and translational research.

**Antonio Lacquaniti, MD**, graduated with vote 110/110 cum laude in medicine and surgery at the University of Messina (Italy) on July 27, 2006 debating an experimental thesis on “Stem Cells in Uremia”. In 2007 he was admitted to the postgraduate school of Nephrology II of the University of Messina. From 2007 to 2012, during his postgraduate medical training in Nephrology, he had several responsibilities in different areas, but in particular in the outpatient management of renal transplant patients (evaluating patients to be inserted on cadaver and living donor transplant waiting lists, follow-ups of transplant recipients), renal biopsy and the histopathological pattern studies of renal disease, and intermittent and continuous dialysis techniques in patients with acute renal failure or with multiorgan failure in the Intensive Care Unit or in other Units. Lacquaniti has also conducted research activities during these years, with the publication in national and international journals of over fifty scientific articles in the nephrology field.

**Stefania Mondello**, MD, MPH, PhD, is a trained neurointensivist with an extensive experience in critical care, biomarker research and statistical analysis methods. She received her medical degree and completed her residency at the University of Messina. After this she obtained her Master’s in Public Health at the University of Florida and then continued studies with a Ph.D. degree focusing on assessments of the clinical utility of brain damage biomarkers to assist in the management of severe traumatic brain injury patients. For the past 5 years she has been attending the Division of Critical Care Medicine at the University of Florida. Her clinical and PhD training led her to also assume the responsibility of Director of Clinical Research at the biotech company, Banyan Biomarkers, Inc. Her research focuses on the use of biochemical markers to improved management, diagnosis and prognosis of patients including clinical validation and assessment of the relationships with clinical variables and physiologic monitoring. These research projects are being carried out in collaboration with NIH and DoD grants. Her intellectual contributions are documented in peer reviewed manuscripts, book chapters, abstracts, and presentations at international scientific meetings. She has been invited to serve on national and international grant review panels and has received a number of awards recognizing her contributions to the field. She has also been recognized by the prestigious Nature Journal.

**Davide Bolignano, MD**, graduated in Medicine at the University of Messina (Italy) in 2004 and obtained the post-graduate specialization in nephrology in 2009, both cum laude. He currently works as clinical researcher of the Italian National Research Council (CNR) at the Institute of Clinical Physiology in Reggio Calabria, Italy. He has participated in several national and international scientific congresses and courses focusing on epidemiology, biostatistics, physiology and pathophysiology of kidney diseases. In 2012, he joined the Cochrane Renal Group (Sydney, Australia) as an honorary research fellow, learning skills in systematic reviews, meta-analysis and literature research. In June 2014 he successfully completed the Global Clinical Research Training Program in Clinical Trials at the Harvard Medical School and currently he is PhD candidate at the Erasmus University in Rotterdam, the Netherlands. Since December 2012 he has been fellow of the European Renal Best Practice group of the ERA-EDTA, providing methodological support for the realization of the forthcoming European guidelines in nephrology. Up to now, he is author of one monograph, author/co-author of 3 book chapters, of over 80 scientific papers published on national and international peer-review journals and of over 70 official interventions (poster presentations, free communications and invited lectures) at national and international congresses on nephrology, metabolism, cardiovascular and laboratory medicine. His main fields of research are: the epidemiology and pathophysiology of chronic and acute kidney diseases, renal biomarkers, cardiovascular risk, vasopressin system, medullar and systemic effects of erythropoietin and stem cells in uremia.

**Vincenzo Pitini,** MD and Chief of “High dose chemotherapy and Stem cell transplantation department” at Universitary Hospital “G. Martino” in Messina. He graduated in Medicine and Surgery in 1977 with 110/110 cum laude. After graduation, he specialized in Renal, haematological diseases, and metabolic disorders in 1980. He specialized in Oncology in 1983. Since August 1980 he has been University Researcher at the Institute of Oncology at the University of Messina, and is still in service at the Department of Human Pathology. Since 1987 he has obtained the qualification of Aid. Since the academic year 1990/91 he has also given lesson cycles on the use of Molecular Biology in Oncology. Since 1995 he has developed the procedures for the collection and subsequent reinfusion of circulating stem cells in the high-dose antiblastic therapy, thereby contributing to the accreditation of the Division of Medical Oncology at the Italian Group for Bone Marrow Transplantation (GITMO) CIC 669. He also attended an updating course in Oncohematology at the University of Texas M.D. Anderson Cancer Center in Houston, Texas (USA). He is also author of scientific papers published on national and international peer-review journals focusing on hematology and oncology fields.

**Carmela Aloisi, MD**, graduated in Medicine and Surgery at the University of Messina in 1975 with vote 110/110 cum laude. She specialized in “Renal, haematological diseases, and metabolic disorders in 1978, in “Anaesthesia and resuscitation” in 1981, in “General Haematology (Clinical and Laboratory)” in 1986. In August 1980 she became University Researcher at the Institute of General Medicine and Medical Care at the University of Messina, and she is still in service at the Department of Internal Medicine. From 1998 to November 2010 she was Manager of the Peritoneal Dialysis service. Since December 2010 she has been Director of the simple unit of Peritoneal Dialysis afferent to the complex unit of Sub-intensive Care and Dialysis Techniques at the Department of Internal Medicine. She also substitutes the Chief of the unit of Sub-intensive Care and Dialysis Techniques when he is absent. She is docent in the Integrated Course of Metabolic, Endocrine, Kidney and Urinary tract Diseases, and in the Integrated Course of Pathophysiology of Human Diseases of the Degree Course in Medicine and Surgery. She also holds many lectureships at several Schools of Specialization at the Faculty of Medicine and Surgery.

**Michele Buemi, MD**, graduated in medicine and surgery in 1973. After graduation in 1974, he became an assistant at the Institute of General Medicine and Medical Care at the University of Messina. He specialized in renal, hematologic diseases, and metabolic disorders in 1976. In 1979 he became university lecturer/professor of renal physiology at the School for Specialization in Nephrology, and, in 1985, lectured on andrologic urology at the School for Specialization in Diabetology. In 1986 he became Associate Professor of Nephrology at the Institute of Internal Medicine at the University of Messina. In 1996 he became Head of the Division of Subintensive Metabolic and Dialytic Care at the Department of Internal Medicine in Messina. In 1998 he became Director of the School of Specialization in Nephrology and Active Member of the Scientific Board of the Center of Clinical Physiology of the CNR (National Research Center) in Reggio Calabria, Italy. In 2010 he became Full Professor of Nephrology. His research interests focus on renal pathophysiology and arterial hypertension.

## Abbreviations

CRP: C Reactive Protein
IL: Interleukin
CACS: Cancer-related anorexia cachexia syndrome
BMI: Body mass index
NYHA: New York Heart Association
ELISA: Enzyme-linked immunosorbent assay
ROC: Receiver operating characteristics
AUC: Area under the curve
ERK 1/2: Extracellular signal-regulated kinases1/2.

## References

[1] Maltoni M, Nanni O, Pirovano M, Scarpi E, Indelli M, Martini C, Monti M, Arnoldi E, Piva L, Ravaioli A, Cruciani G, Labianca R, Amadori D (1999). Successful validation of the palliative prognostic score in terminally ill cancer patients. Italian multicenter study group on palliative care. J Pain Symptom Manage.

[2] Strasser F, Bruera ED (2002). Update on anorexia and cachexia. Hematol Oncol Clin North Am.

[3] Lacquaniti A, Altavilla G, Picone A, Donato V, Chirico V, Mondello P, Aloisi C, Marabello G, Loddo S, Buemi A, Lorenzano G, Buemi M (2014). Apelin beyond kidney failure and hyponatremia: a useful biomarker for cancer disease progression evaluation. Clin Exp Med.

[4] Berenstein EG, Ortiz Z (2004). Megestrol acetate for the treatment of anorexia-cachexia syndrome (Protocol for a Cochrane Review). Cochrane Libr.

[5] Baiti NB, Walsh D (2009). What is cancer anorexia-cachexia syndrome? A historical perspective. J R Coll Physicians Edinb.

[6] Evans WJ, Morley JE, Argiles J, Bales C, Baracos V, Guttridge D, Jatoi A, Kalantar-Zadeh K, Lochs H, Mantovani G, Marks D, Mitch WE, Muscaritoli M, Najand A, Ponikowski P, Rossi Fanelli F, Schambelan M, Schols A, Schuster M, Thomas D, Wolfe R, Anker SD (2008). Cachexia: a new definition. Clin Nutr.

[7] Ferguson M, Isenring E, Bauer J, Banks M, Capra S (2008). An amendment to the 2002 ASPEN Guideline Statements. Nutr Clin Pract.

[8] Huhmann MB, August DA (2008). Review of American Society for Parenteral and Enteral Nutrition (ASPEN) Clinical Guidelines for Nutrition Support in Cancer Patients: nutrition screening and assessment. Nutr Clin Pract.

[9] Klein S, Kinney J, Jeejeebhoy K, Alpers D, Hellerstein M, Murray M, Twomey P (1997). Nutrition support in clinical practice: review of published data and recommendations for future research directions. Summary of a conference sponsored by the National Institutes of Health, American Society for Parenteral and Enteral Nutrition, and American Society for Clinical Nutrition. Am J Clin Nutr.

[10] Laviano A, Gleason JR, Meguid MM, Yang ZJ, Cangiano C, Rossi Fanelli F (2000). Effects of intra-VMN mianserin and IL-1ra on meal number in anorectic tumor-bearing rats. J Investig Med.

[11] Garcia JM, Garcia-Touza M, Hijazi RA, Taffet G, Epner D, Mann D, Smith RG, Cunningham GR, Marcelli M (2005). Active ghrelin levels and active to total ghrelin ratio in cancer-induced cachexia. J Clin Endocrinol Metab.

[12] Utech AE, Tadros EM, Hayes TG, Garcia JM (2012). Predicting survival in cancer patients: the role of cachexia and hormonal, nutritional and inflammatory markers. J Cachexia Sarcopenia Muscle.

[13] Lacquaniti A, Bolignano D, Campo S, Perrone C, Donato V, Fazio MR, Buemi A, Sturiale A, Buemi M (2009). Malnutrition in the elderly patient on dialysis. Ren Fail.

[14] Fouladiun M, Korner U, Bosaeus I, Daneryd P, Hyltander A, Lundholm KG (2005). Body composition and time course changes in regional distribution of fat and lean tissue in unselected cancer patients on palliative care—correlations with food intake, metabolism, exercise capacity, and hormones. Cancer.

[15] Yeh SS, Hafner A, Chang CK, Levine DM, Parker TS, Schuster MW (2004). Risk factors relating blood markers of inflammation and nutritional status to survival in cachectic geriatric patients in a randomized clinical trial. J Am Geriatr Soc.

[16] Bolignano D, Coppolino G, Donato V, Lacquaniti A, Bono C, Buemi M (2010). Neutrophil gelatinase-associated lipocalin (NGAL): a new piece of the anemia puzzle?. Med Sci Monit.

[17] Mantovani G, Macciò A, Mura L, Massa E, Mudu MC, Mulas C, Lusso MR, Madeddu C, Dessì A (2000). Serum levels of leptin and proinfiammatory cytokines in patients with advanced stage cancer at different sites. J Mol Med.

[18] Weryńska B, Kosacka M, Gołecki M, Jankowska R (2009). Leptin serum levels in cachectic and non-cachectic lung cancer patients. Pneumonol Alergol Pol.

[19] Wren AM, Bloom SR (2007). Gut hormones and appetite control. Gastroenterology.

[20] Neary NM, Small CJ, Wren AM, Lee JL, Druce MR, Palmieri C, Frost GS, Ghatei MA, Coombes RC, Bloom SR (2004). Ghrelin increases energy intake in cancer patients with impaired appetite: acute, randomized, placebo-controlled trial. J Clin Endocrinol Metab.

[21] Lacquaniti A, Donato V, Chirico V, Buemi A, Buemi M (2011). Obestatin: an interesting but controversial gut hormone. Ann Nutr Metab.

[22] Lacquaniti A, Donato V, Chirico V, Pettinato G, Buemi M (2011). From chronic kidney disease to transplantation: the roles of obestatin. Regul Pept.

[23] Lacquaniti A, Bolignano D, Donato V, Chirico V, Romeo A, Loddo S, Buemi M (2011). Obestatin: a new element for mineral metabolism and inflammation in patients on hemodialysis. Kidney Blood Press Res.

[24] Ren AJ, Guo ZF, Wang YK, Wang LG, Wang WZ, Lin L, Zheng X, Yuan WJ (2008). Inhibitory effect of obestatin on glucose-induced insulin secretion in rats. Biochim Biophys Res Commun.

[25] Samson WK, Yosten G, Chang JK, Ferguson AV, White MM (2008). Obestatin inhibits vasopressin secretion: evidence for a physiological action in the control of fluid homeostasis. J Endocrinol.

[26] Nakahara T, Harada T, Yasuhara D, Shimada N, Amitani H, Sakoguchi T, Kamiji MM, Asakawa A, Inui A (2008). Plasma obestatin concentrations are negatively correlated with body mass index, insulin resistance index, and plasma leptin concentrations in obesity and anorexia nervosa. Biol Psychiatry.

[27] Gioulbasanis I, Georgoulias P, Vlachostergios PJ, Baracos V, Ghosh S, Giannousi Z, Papandreou CN, Mavroudis D, Georgoulias V (2011). Mini Nutritional Assessment (MNA) and biochemical markers of cachexia in metastatic lung cancer patients: interrelations and associations with prognosis. Lung Cancer.

[28] Suzuki H, Asakawa A, Amitani H, Nakamura N, Inui A (2013). Cancer cachexia–pathophysiology and management. J Gastroenterol.

[29] Nagaya N, Moriya J, Yasumura Y, Uematsu M, Ono F, Shimizu W, Ueno K, Kitakaze M, Miyatake K, Kangawa K (2004). Effects of ghrelin administration on left ventricular function, exercise capacity, and muscle wasting in patients with chronic heart failure. Circulation.

[30] Kvist Reimer M, Pacini G, Ahrén B (2003). Dose-depent inhibition by ghrelin of insulin secretion in mouse. Endocrinology.

[31] Saad MF, Bernarba B, Hwu CM, Jinagouda S, Fahmi S, Kogosov E, Boyadjian R (2002). Insulin regulates plasma ghrelin concentration. J Clin Endocrinol Metabol.

[32] Broglio F, Gottero C, Benso A, Prodam F, Destefanis S, Gauna C, Maccario M, Deghenghi R, van der Lely AJ, Ghigo E (2003). Effect of ghrelin on the insulin and glycemic responces to glucose, arginine or free fatty acids load in humans. J Clin Endocrinol Metabol.

[33] Bing C, Taylor S, Tisdale MJ, Williams G (2001). Cachexia in MAC16 adenocarcinoma: suppression of hunger despite normal regulation of leptin, insulin and hypothalamic neuropeptide Y. J Neurochem.

[34] Alloatti G, Arnoletti E, Bassino E, Penna C, Perrelli MG, Ghé C, Muccioli G (2010). Obestatin affords cardioprotection to the ischemic-reperfused isolated rat heart and inhibits apoptosis in cultures of similarly stressed cardiomyocytes. Am J Physiol Heart Circ Physiol.

[35] Egido EM, Hernández R, Marco J, Silvestre RA (2009). Effect of obestatin on insulin, glucagon and somatostatin secretion in the perfused rat pancreas. Regul Pept.

[CR36] The pre-publication history for this paper can be accessed here:http://www.biomedcentral.com/1471-2407/14/828/prepub

